# High Incidence of Benign Perianal Disorders After Sleeve Gastrectomy and One Anastomosis Gastric Bypass

**DOI:** 10.1007/s11695-025-08342-1

**Published:** 2025-10-18

**Authors:** Eyal Yonathan Juster, Raja Magdoub, Amram Kupietzky, Bilal Aliyan, Ata Maden, Ronit Grinbaum, Noam Shussman, Haggi Mazeh, Ido Mizrahi

**Affiliations:** 1 Department of Surgery, Hadassah Medical Center, Jerusalem, Israel; 2 Faculty of Medicine, Hebrew University of Jerusalem, Jerusalem, Israel

**Keywords:** Benign perianal disorders, Obesity, Bariatric surgery, Hemorrhoidal disease, Anal fissure, Perianal abscess, Fistulae

## Abstract

**Aim:**

Bariatric surgery may alter bowel habits, potentially leading to new-onset benign perianal disorders (NOPD). This study aimed to assess the incidence and identify potential risk factors of NOPD following vertical sleeve gastrectomy (VSG) and one anastomosis (mini) gastric bypass (OAGB).

**Methods:**

We conducted a retrospective cross-sectional, single-center study using retrospective analysis of prospectively collected data. All patients who underwent VSG or OAGB between 2015–2018 were considered. NOPD diagnoses were based on physical examinations and a standardized phone questionnaire assessing past and present perianal symptoms.

**Results:**

Of 540 eligible patients, 313 participated (150 VSG, 163 OAGB). Among 264 patients without prior perianal disorders, 96 (36.4%) developed NOPD postoperatively—29.6% in the VSG group vs. 42.4% in the OAGB group (*p* = 0.04). The most common conditions were hemorrhoidal disease (24.2%), fissures (12.8%), and abscesses/fistulas (3%). Surgical treatment was required in 17.7% (17/96) of affected patients. Increased bowel movement frequency was more common after OAGB than VSG (57.7% vs. 11.7%, *p* < 0.001). OAGB, younger age, and altered bowel habits were associated with higher NOPD risk. Forty-nine patients (15.7%) reported preexisting perianal disorders, 26.5% of whom experienced worsening symptoms. Overall, 86% of patients were unaware of the link between bariatric surgery and NOPD, and 15% indicated they would not recommend surgery due to these symptoms.

**Conclusion:**

NOPD are relatively common after bariatric surgery, particularly OAGB. Patients—especially those with identified risk factors—should be counselled regarding this potential complication during the preoperative decision-making process.

**Supplementary Information:**

The online version contains supplementary material available at 10.1007/s11695-025-08342-1.

## Introduction

The number of bariatric surgeries performed worldwide has increased drastically by more than tenfold in the last two decades [1]. Bariatric surgery has been found to be the most effective treatment of obesity to date. Conservative medical treatments have proven inefficient in maintaining long-term weight loss and preventing comorbidities, in comparison to bariatric surgery [2]. Among the currently available bariatric surgeries, the Roux-en-Y gastric bypass (RYGB) prevents long-term weight gain and reduces the burden of obesity related comorbidities [3]. The one anastomotic gastric bypass (OAGB), despite being a newer procedure, demonstrates results that are thought to be equal if not superior to RYGB [4]. Importantly, the OAGB has become the most popular bariatric procedure performed in Israel accounting for 62% of surgeries in recent years [5]. The third common procedure is sleeve gastrectomy with early and long-term results that are inferior to both the above-mentioned gastric bypasses [6–8].

The different bariatric surgeries can lead to changes in bowel habits as well as gastrointestinal symptoms such as nausea and vomiting, heartburn and dumping syndrome, irrespective of the specific sub-type of bariatric surgery performed [9–11]. For example, after restrictive bariatric surgery such as sleeve gastrectomy, new onset constipation was reported among 40% of patients, most likely due to the restriction of fluid intake affecting stool consistency and texture [12]. On the contrary, following gastric bypass procedures which primarily rely on malabsorptive mechanisms, nearly 46% of patients have reported diarrhea [13]. Other mechanisms that might cause post operative diarrhea after bypass surgeries include shortening of the absorptive bowel loop, changes in the gastrointestinal microbiome, protein losing enteropathies, bile acid malabsorption, intra operative vagal nerve injury, and dumping syndrome caused by rapid delivery of undigested food to the small intestine [13].

The abovementioned gastrointestinal symptoms might result in significant postoperative discomfort among patients and affect their quality of life. Furthermore, pathological bowel habits including prolonged constipation and diarrhea were associated with an increased incidence of post operative perianal disorders such as hemorrhoidal disease, anal fissures, and perianal abscesses [14].

To the best of our knowledge, only four original studies have been published on this topic so far [14–17]. The studies were limited to relatively small patient cohorts and did not study the incidence of anal disorders after one-anastomosis gastric bypass. Furthermore, the studies show varying incidence of new onset benign perianal disorders (3.8%−37%) following bariatric surgery.

Due to the insufficient and contradicting evidence on this issue, the aim of this study was to further assess the incidence and identify potential risk factors of new onset benign perianal disorders following two of the most common bariatric surgeries performed in Israel and worldwide: sleeve gastrectomy and one-anastomosis gastric bypass, among a large cohort of patients.

## Methods

A retrospective analysis of prospectively collected data was performed to identify all consecutive patients who underwent either laparoscopic vertical sleeve gastrectomy (VSG), or laparoscopic one anastomosis (mini) gastric bypass (OAGB) at our institution. To reach a long-term follow up of at least 5 years, we included patients who underwent surgery between 2015 and 2018. Data extraction included patient demographics, operative and postoperative outcomes, and weight loss outcomes. All patients were contacted via telephone and were offered to participate in the study. Patients who agreed to participate were presented with a structured phone questionnaire focusing on perianal disorders (appendix 1).

A new onset perianal disorder was considered if the patient reported having one of the above: hemorrhoidal disease; anal fissure; perianal abscess and/or fistulae; and fecal incontinence. Patients who reported having a benign perianal disorder prior to their bariatric surgery were asked for worsening of their specific perianal condition or for the appearance of a new perianal disorder. Self-reported conditions prior to their bariatric surgery were considered irrespective of when they had occurred, and patients with a history of anorectal disease were excluded from the primary analysis.

Constipation and diarrhea were assessed through patient self-reporting via a postoperative questionnaire. Constipation was defined as the presence of symptoms consistent with the Rome IV criteria, including infrequent bowel movements (fewer than three per week), straining during defecation, sensation of incomplete evacuation, or hard/lumpy stools. Diarrhea was defined as the passage of loose or watery stools occurring more than three times per day, in line with standard clinical definitions.

All data were checked against the electronic medical record both in-hospital and via the health care provider online record system to minimize recall bias. Patients’ records reporting new-onset perianal disorders were reviewed with specific attention to the documentation of their specific disorder by a colorectal surgeon. Patients were not systematically referred for colorectal evaluation as part of the study protocol. Typical symptoms documented in the clinical records included anal pain, bleeding, pruritus ani, discharge, and changes in bowel habits such as persistent constipation or diarrhea.

The Institutional Review Board approved the study, and informed consent was obtained from patients who agreed to participate in the study. Exclusion criteria were patients under the age of 18 at the time of the operation; patients who could not be reached; patients who refused to participate; patients who despite reporting a perianal disorder did not have a documented colorectal surgeon visit supporting their complaint; and patients who did not complete the interview or questionnaire. This study was conducted and reported in accordance with the Strengthening the Reporting of Observational Studies in Epidemiology (STROBE) guidelines.

Our operative technique for OAGB includes resection of the body and fundus of the stomach, thus constructing a sleeve gastrectomy pouch over a 36-french bougie. Subsequently, an anastomosis is performed between the gastric pouch to the jejunum at a point identified 200 cm’s distal to the ligament of Treitz. Our operative technique for sleeve gastrectomy includes resection of the body and fundus of the stomach over a 36-french bougie. All surgeries were performed by an expert bariatric surgeon.

All patients underwent preoperative assessments in our multidisciplinary bariatric clinic and met the previously proposed criteria of the performance of bariatric surgery [18]. A detailed dialogue was held with patients regarding the available surgical options including laparoscopic VSG, OAGB, and RYGB, covering the pros and cons of each procedure as well as potential short- and long-term complications. Patients with clinically symptomatic gastroesophageal reflux disease were advised against VSG or OAGB and were counseled in favor of RYGB. Patients with comorbid metabolic syndrome, particularly those with type 2 diabetes mellitus, were advised to consider RYGB or OAGB, as these procedures tend to offer improved resolution of comorbidities compared with VSG [19]. bowel habits were not routinely discussed in the preoperative clinic.

Sample size estimation for this research aimed to ensure adequate precision to estimate the incidence of perianal disorders among patients undergoing bariatric surgery. With an estimated target population of approximately 300 patients and an expected incidence rate of 37%, based on the results of the most recent and relevant study by Cano-Valderrama et al., the calculation yielded that a sample size of 300 participants results in a 95% confidence interval ranging from 31.5% to 42.7%. Data collection was concluded once the final cohort reached a total of 540 patients, thus surpassing the necessary sample size estimated above.

All statistical analyses were reviewed by a statistician. Continuous variables were expressed as mean, median, and standard deviation. Association between two categorical variables was performed by using the Chi-square test or the Fisher’s exact test. Quantitative variables were compared between two-independent group by using the two-sample t-test. To simultaneously assess the impact of multiple independent variables on the dichotomous dependent variable, we employed a multivariable logistic regression model. The comparison of the prevalence of a categorical variable to the literature was conducted using a one-sample Chi-square test. All statistical tests were two-tailed, and a p-value of less than 0.05 was considered statistically significant.

## Results

**Fig. 1:**
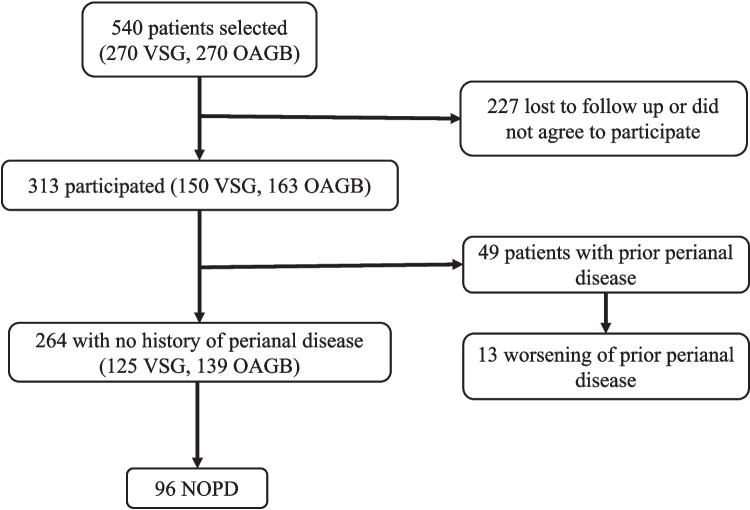
Patient flow chart. OAGB one anastomosis gastric bypass, VSG Vertical sleeve gastrectomy, NOPD new onset perianal disorder

During the study period, a total of 540 patients underwent either laparoscopic OAGB or VSG; however, only 313 consented to participate in the study and answered the structured phone questionnaire (Fig. 1). Notably, no patients were excluded due to lack of colorectal surgeon documentation, as all cases of reported NOPD were validated through electronic medical records and specialist confirmation. Among these patients, the mean age was 39.4 and 203 (64.9%) were females. VSG was performed in 150 (47.9%) and OAGB in 163 163 (52.1%) patients.

Patients’ demographic and clinical features are presented in Table 1. The groups were comparable for preoperative hypertension (HTN) and obstructive sleep apnea (OSA); however, preoperative diabetes mellitus was significantly more prevalent in the OAGB group (26.9% vs. 14.8%, *p* = 0.009). Patients in the OAGB group were older at the time of surgery compared to patients in the VSG group (mean age 42.5 ± 10.5 vs. 36.6 ± 11 years, *p* < 0.001). Additionally, OAGB was performed more often as revisional surgery compared to VSG (*p* < 0.001).

**Table 1:** Patients demographics

	All *N* = 313	OAGB *N* = 163	VSG *N* = 150	*p* value
Age (mean) years	39.4 ± 11.1	42.5 ± 10.6	36.6 ± 11.8	< 0.001
Female Gender	203 (65%)	101 (62%)	102 (68%)	0.26
Pre-op weight (mean) Kg	121.5 ± 21.3	122.6 ± 22.3	120.6 ± 20.3	0.4
Pre-op BMI (mean)	43.3 ± 5.84	43.6 ± 6.01	42.9 ± 5.65	0.283
Diabetes mellitus	65 (20.1%)	43 (26.4%)	22 (14.7%)	0.009
HTN	67 (21.4%)	38 (23.3%)	29 (19.3%)	0.36
Prior perianal disorder	49 (15.6%)	24 (14.7%)	25 (16.7%)	0.63
Prior bariatric surgery	46 (14.6%)	44 (26.9%)	2 (1.3%)	< 0.001
VSG	27 (58.7%)	27 (61.3%)	0	-
LAGB	21 (45.6%)	19 (43.1%)	2	-

*OAGB* one anastomosis gastric bypass, *VSG* Vertical sleeve gastrectomy, *BMI* body mass index, *OSA *obstructive sleep apnea, *HTN* hypertension, *LAGB* Laparoscopic Adjustable Gastric Banding

Postoperative data was compared between the two study groups (Table 2). Postoperative mean BMI was lower in the OAGB group (BMI 28.4 ± 5.3 vs. BMI 29.7 ± 5.1, *p* = 0.023). A significantly higher number of patients who underwent OAGB reported postoperative changes in bowel habits (*p* < 0.001). Specifically, OAGB patients reported having a significantly higher frequency of daily bowel movements (2.6 ± 1.98 vs. 1.1 ± 0.8 per day, *p* < 0.001) and diarrhea (18.4% vs. 8%, *p* < 0.001). Conversely, patients who underwent VSG reported having higher rates of postoperative constipation compared to patients who underwent OAGB (20% vs. 6%, *p* < 0.001).

**Table 2:** Post operative variables

	OAGB *N* = 163	VSG *N* = 150	*p *value
Follow up time (mean) months	57.7 ± 17.3	71.6 ± 13.5	< 0.001
Postoperative weight (mean, Kg)	80.06 ± 18.6	83.56 ± 16.5	0.081
Postoperative BMI (mean)	28.4 ± 5.3	29.7 ± 5.1	0.023
Bowel movements (per day)	2.6 ± 1.98	1.1 ± 0.8	< 0.001
Any Postoperative changes in bowel habits	102 (62.6%)	54 (36.7%)	< 0.001
More frequent bowel movements	94 (57.7%)	17 (11.7%)	< 0.001
Constipation	11 (6.7%)	31 (20.7%)	< 0.001
Diarrhea	90 (55.2%)	12 (8%)	< 0.001

*OAGB* one anastomosis gastric bypass, *VSG* Vertical sleeve gastrectomy,* BMI* body mass index, changes in bowel habits: more frequent, less frequent, softer or firmer stools

Forty-nine (15.6%) patients had prior history of a perianal disorder and therefore were not considered as having a new-onset perianal disorder. Of these, thirteen (21.6%) patients reported worsening of their preexisting condition. After excluding patients with prior history of perianal disorder, 264 patients were included in the final analysis: 125 (47.3%) patients who underwent VSG and 139 (52.7%) patients who underwent OAGB.

After a mean follow-up time of 63 ± 17 months, 96 (36.4%) of patients without a history of prior perianal disorder developed a new onset perianal disorder (NOPD).

The incidence of NOPD was significantly higher among patients who underwent OAGB (59/139, 42.4%) compared to patients who underwent VSG (37/125, 29.6%), (*p* = 0.04). The odds ratio for developing NOPD in OAGB patients compared to those who underwent VSG was 1.75 (95% CI: 1.05–2.92).

The most common NOPD reported was hemorrhoidal disease (24.2%), followed by anal fissure (12.8%), perianal abscess & fistulae (3%), and fecal incontinence (3%). Among patients who reported having NOPD, 17 (6%) patients required surgical intervention for their NOPD; left lateral anal sphincterotomy for anal fissure (*n* = 10), hemorrhoidectomy (*n* = 6), and abscess & fistula drainage with seton placement (*n* = 5). While hemorrhoidal disease and anal fissures were the most common NOPDs across all bariatric patients, there was no significant association between the type of surgery and the risk of developing hemorrhoidal disease or anal fissure. Conversely, patients who underwent OAGB had a significantly higher risk of developing fecal incontinence (5.7% vs. 0.8%, *p* = 0.03) compared to those who underwent VSG (Table 3).

**Table 3:** Patients with New Onset Perianal Disorder

	All *N* = 264	VSG *N* = 125	OAGB *N* = 139	*p* value
New onset perianal disorder	*96 (36.3%)*	37 (29.6%)	59 (42.4%)	0.03
Hemorrhoidal disease	64 (24.2%)	25 (20%)	39 (28%)	0.09
Anal fissure	34 (12.8%)	11 (8%)	23 (16.5%)	0.06
Perianal abscess & fistulae	9 (3%)	3 (2.4%)	6 (4.3%)	0.39
Fecal incontinence	9 (3%)	1 (0.8%)	8 (5.7%)	0.03
Surgical intervention for NOPD	17 (6.4%)	8 (6.4%)	9 (6.4%)	0.98

*OAGB* one anastomosis gastric bypass, *VSG* Vertical sleeve gastrectomy, *NOPD* new onset perianal disorder

Patients who developed NOPD were younger at the time of surgery (mean age 37.8 ± 10.4 vs. 40.5 ± 11.9 years, *p* = 0.05). The incidence of NOPD was higher among patients who experienced postoperative change in bowel habits (52% vs. 17.2%, *p* < 0.001). Univariate analysis demonstrated three variables that were associated with increased risk for developing NOPD: type of surgery (OAGB), younger age at the time of surgery; and postoperative changes in bowel habits. Table 4 presents risk stratification for the development of NOPD.

**Table 4:** Patients’ variables and association with the risk of developing NOPD – Univariate analysis

All = 264	NOPD *N* = 96	No NOPD *N* = 168	*p* value
Female gender	61 (63.5%)	113 (67.3%)	0.66
OSA	18 (18.7%)	25 (14.9%)	0.43
DM	15 (15.6%)	34 (20.2%)	0.34
HTN	20 (20.8%)	37 (22.0%)	0.8
Surgery type (OAGB)	59 (61.4%)	80 (47.6%)	0.04
Age at surgery (mean) years	37.8 ± 10.4	40.5 ± 11.9	0.05
Pre-op BMI	43.7 ± 6.8	43.1 ± 5.3	0.43
Changes in bowel habits	67 (69.7%)	29 (17.2%)	< 0.001
Post-op BMI	29.13 ± 6.1	29.02 ± 4.8	0.87

*NOPD* New onset perianal disorder, *VSG* Vertical sleeve gastrectomy, *BMI* body mass index, *OSA* obstructive sleep apnea, *HTN* hypertension

Multivariate analysis using a logistic regression model demonstrated that older age at the time of surgery was associated with a decreased risk for developing NOPD (adjusted OR 0.97, 95% CI = 0.95–0.99, *P* = 0.04). Postoperative change in bowel habits was associated with an increased risk for developing NOPD (adjusted OR 4.00, 95% CI = 2.29–7.00, *p* < 0.001). However, surgery type (OAGB versus VSG) was not statistically significant as a potential risk factor for developing NOPD (adjusted OR 1.52, 95% CI = 0.855–2.704, *p* = 0.154). See Table 5.

**Table 5:** Multivariate analysis of risk factors for developing NOPD

	Adjusted OR	95% Confidence Interval	*p* value
Surgery type (OAGB)	1.52	0.855–2.704	0.154
Older age at surgery	0.974	0.950–0.999	0.043
Post-op changes in bowel habits	4.00	2.292–7.00	< 0.001

*OAGB *one anastomosis gastric bypass, changes in bowel habits: more frequent, less frequent, softer or firmer stools

Most patients (86%) were not aware of the association between bariatric surgery and the risk of developing NOPD, and 15% of patients answered they would not recommend bariatric surgery to other patients due to the new onset or worsened perianal disorder they experienced following their operation.

## Discussion

The results of our study demonstrate a high incidence (36.4%) of new onset benign perianal disorder (NOPD) following bariatric surgery. Our study consists of the largest cohort of patients published on NOPD to date and is essentially the only study on NOPD following the increasingly popular OAGB.

Obesity is increasingly recognized as a contributing factor in the development of benign anorectal pathologies. García-Redondo et al. demonstrated a high prevalence of conditions such as hemorrhoidal disease and anal fissures among patients evaluated for bariatric surgery, underscoring the burden of anorectal disease in this population [20]. Complementing these clinical observations, Huang et al. employed Mendelian randomization to establish a causal link between adiposity traits, including BMI, body fat percentage, and waist circumference, and the risk of hemorrhoidal disease, reinforcing the biological plausibility of this association [21]. In our study, the prevalence of prior anorectal disease was 15.6%, based on patient self-reports collected via a postoperative questionnaire. These individuals were excluded from further analysis. While self-reported data may be subject to recall bias and potential underestimation, patients typically remember clinically significant symptoms, especially those that caused discomfort or required medical intervention.

Paradoxically, our findings, together with recent reports from other groups, suggest that bariatric surgery may exacerbate these conditions rather than alleviate them. Previous studies demonstrated varying incidence of new onset benign perianal disorders (3.8%−37%) after bariatric surgery [14–17]*.* The first study was published in 2008 and examined the incidence of benign perianal disorders following biliopancreatic diversion (BD) surgeries among super-obese patients [17]. After a follow-up time of 5 years, 18% of patients presented with NOPD, with anal fissure being the most common complication (8%). Another study examined the efficacy of botulinum toxin injection therapy as a treatment for chronic anal fissure among fifty-nine patients who underwent biliopancreatic diversion [16]. In this study, the authors chose botulinum toxin over lateral internal sphincterotomy (which is considered the standard surgical treatment for anal fissure), because a sphincterotomy might cause fecal incontinence among patients suffering from severe diarrhea. The success rate of botulinum toxin injection therapy was 59%, with one patient developing a transient episode of fecal incontinence. The third study by Cano-Valderrama et al. included 196 patients who underwent either gastric bypass or modified biliopancreatic diversion [14]. Approximately 37% of patients developed a new onset post operative perianal disorder; hemorrhoidal disease 16.1%, anal fissure 10.2%, and abscess & fistulae 5.8%. Inconsistently with the incidence presented above, a recent study by Salgado-Nesme et al. published in 2020, demonstrated an NOPD incidence of 3.8% (9/235 patients) among patients who underwent either vertical sleeve gastrectomy or Roux en Y gastric bypass [17]. Our study demonstrates that a significantly higher number of patients after OAGB developed NOPD compared to patients after VSG (42.4% vs 29.6%, p = 0.03). Since our study is the only study including patients after OAGB, a comparison to other studies is impossible. Additionally, a significant association was found between a higher frequency of postoperative bowel habits and the risk of developing NOPD. This may be the explanation for the higher incidence of NOPD among patients who underwent OAGB, as patients after OAGB reported having a higher frequency of postoperative bowel habits compared to patients who underwent VSG (62% vs. 36%). Similarly, Cano-Valderrama et al. identified that the incidence of NOPD was higher after modified biliopancreatic diversion compared to gastric bypass surgery, attributing this difference to the higher frequency of bowel habits in patients after biliopancreatic diversion [14].

Univariate analysis suggested that bariatric surgery type (OAGB versus VSG), younger age at the time of surgery, postoperative changes in bowel habits (diarrhea, constipation, and changes in stool consistency) were all associated with an increased risk of developing NOPD. However, multivariate analysis indicated that the most significant risk factor for developing NOPD after bariatric surgery is the presence of postoperative alterations in bowel habits. These findings emphasize the importance of postoperative monitoring of bowel habits, regardless of the type of surgery, as these changes appear to play a more critical role in the risk of developing NOPD. Patients after bariatric surgery should be asked about their bowel habits and counselled appropriately for optimal defecatory habits.

In our study, 17.7% of patients (17/96) who developed NOPD after bariatric surgery required surgical treatment for their perianal disorder. Similarly, Cano-Valderrama et al. reported that 27% of patients (14/51) who developed NOPD after gastric bypass or modified biliopancreatic diversion required surgical intervention [14]. However, it is essential to remember that even patients with NOPD who are managed successfully with conservative treatment alone, complain of pain, discomfort, defecatory abnormalities, and consequently complain of a decrease in their quality of life [22, 23].

Patient education on the association between NOPD and bariatric surgery is quite low as shown by the two final questions on our phone survey. First, most patients (86.6%) were not aware of any possible association between bariatric surgery and perianal disorders. Second, forty-seven (15%) patients reported that they would not recommend bariatric surgery to other patients due to their fear of developing NOPD. Importantly, 49 patients (15%) reported having a perianal disorder prior to surgery, and 13 of these patients (26.5%) reported worsening of their prior disorder. These points emphasize the importance of preoperative patient education on all aspects of bariatric surgery with specific attention given to malabsorptive procedures such as OAGB. Notably, contemporary management of bariatric patients increasingly relies on multidisciplinary care models. Collaboration among bariatric surgeons, gastroenterologists, colorectal surgeons, dietitians, and primary care providers is essential for optimizing postoperative outcomes. Awareness of the unique anorectal disease patterns that can emerge following bariatric surgery is crucial for informed diagnosis, targeted treatment, and appropriate referral pathways within these teams. Perhaps future investigations on this subject would solidify informing bariatric candidates of this potential postoperative complication. In particular, educational strategies aimed at optimizing postoperative bowel habits may help mitigate the risk of NOPD and warrant further study.

The retrospective nature of this study carries an inherent risk of selection bias and recall bias. Additionally, the use of telephone interviews for follow-up introduces the possibility of reporting bias, as only 58% of the eligible cohort ultimately participated. While this may limit generalizability, we acknowledge that non-responders may differ in their prevalence of perianal disorders potentially lower due to absence of symptoms or higher due to reluctance in disclosing sensitive conditions. To mitigate this, we cross-validated patient-reported outcomes with electronic medical records and colorectal surgeon documentation, thereby enhancing the reliability of the data and reducing the impact of potential bias. Perianal disorders are also common in the general population, and although we excluded patients with a history of such disorders, background prevalence may still confound the true effect of bariatric surgery. Furthermore, our comparison was restricted to VSG and OAGB, the most frequently performed procedures in our country. We acknowledge that comparisons with RYGB would be highly informative, particularly given the similar malabsorptive mechanisms between OAGB and RYGB, but this was not feasible in our cohort. Despite these limitations, our study provides initial evidence on the incidence of NOPD after one-anastomosis gastric bypass, which has become a popular bariatric procedure worldwide.

In conclusion, multiple risk factors for developing NOPD were identified and should be considered during the pre and postoperative patient counselling. Further research is needed to develop and assess evidence-based strategies to reduce the incidence of NOPD following bariatric surgery.

## Supplementary Information

Below is the link to the electronic supplementary material.

Supplementary file1 (DOCX 15 KB)

## Data Availability

Data supporting the findings of this study are available from the corresponding author upon reasonable request.

## References

[1] Angrisani L, Santonicola A, Iovino P, Vitiello A, Zundel N, Buchwald H, et al. Bariatric surgery and endoluminal procedures: IFSO worldwide survey 2014. Obes Surg. 2017;27(9):2279–89. 28405878 10.1007/s11695-017-2666-x PMC5562777

[2] Kloock S, Ziegler CG, Dischinger U. Obesity and its comorbidities, current treatment options and future perspectives: challenging bariatric surgery? Pharmacol Ther. 2023;251:108549. 37879540 10.1016/j.pharmthera.2023.108549

[3] Parikh M, Eisenberg D, Johnson J, El-Chaar M. American society for metabolic and bariatric surgery review of the literature on one-anastomosis gastric bypass. Surg Obes Relat Dis. 2018;14(8):1088–92. 29907540 10.1016/j.soard.2018.04.017

[4] Haddad A, Bashir A, Fobi M, Higa K, Herrera MF, Torres AJ, et al. The IFSO worldwide one anastomosis gastric bypass survey: techniques and outcomes? Obes Surg. 2021;31(4):1411–21. 33517557 10.1007/s11695-021-05249-5

[5] Abu-Abeid A, Yuval JB, Keidar A, Nizri E, Lahat G, Eldar SM. Technical considerations in one anastomosis gastric bypass-the Israeli Society of Metabolic and Bariatric Surgery experience. Obes Surg. 2024;34(7):2356–62. 38649670 10.1007/s11695-024-07223-3 PMC11217076

[6] Golomb I, Ben David M, Glass A, Kolitz T, Keidar A. Long-term metabolic effects of laparoscopic sleeve gastrectomy. JAMA Surg. 2015;150(11):1051–7. 26244446 10.1001/jamasurg.2015.2202

[7] Schauer PR, Bhatt DL, Kirwan JP, Wolski K, Aminian A, Brethauer SA, et al. Bariatric surgery versus intensive medical therapy for diabetes - 5-year outcomes. N Engl J Med. 2017;376(7):641–51. 28199805 10.1056/NEJMoa1600869 PMC5451258

[8] Benaiges D, Flores-Le-Roux JA, Pedro-Botet J, Ramon JM, Parri A, Villatoro M, et al. Impact of restrictive (sleeve gastrectomy) vs hybrid bariatric surgery (Roux-en-Y gastric bypass) on lipid profile. Obes Surg. 2012;22(8):1268–75. 22544352 10.1007/s11695-012-0662-8

[9] Potoczna N, Harfmann S, Steffen R, Briggs R, Bieri N, Horber FF. Bowel habits after bariatric surgery. Obes Surg. 2008;18(10):1287–96. 18327626 10.1007/s11695-008-9456-4

[10] Tack J, Arts J, Caenepeel P, De Wulf D, Bisschops R. Pathophysiology, diagnosis and management of postoperative dumping syndrome. Nat Rev Gastroenterol Hepatol. 2009;6(10):583–90. 19724252 10.1038/nrgastro.2009.148

[11] El-Hadi M, Birch DW, Gill RS, Karmali S. The effect of bariatric surgery on gastroesophageal reflux disease. Can J Surg. 2014;57(2):139–44. 24666452 10.1503/cjs.030612 PMC3968207

[12] Goldenshluger M, Goldenshluger A, Keinan-Boker L, Cohen MJ, Ben-Porat T, Gerasi H, et al. Postoperative outcomes, weight loss predictors, and late gastrointestinal symptoms following laparoscopic sleeve gastrectomy. J Gastrointest Surg. 2017;21(12):2009–15. 28971291 10.1007/s11605-017-3585-9

[13] Borbély YM, Osterwalder A, Kröll D, Nett PC, Inglin RA. Diarrhea after bariatric procedures: diagnosis and therapy. World J Gastroenterol. 2017;23(26):4689–700. 28765690 10.3748/wjg.v23.i26.4689 PMC5514634

[14] Cano-Valderrama O, Sánchez-Pernaute A, Rubio MA, Talavera P, Martín-Antona E, Torres AJ. Incidence of new-onset benign anal disorders after bariatric surgery. Clin Obes. 2018;8(1):50–4. 29110411 10.1111/cob.12228

[15] ElíaGuedea M, GraciaSolanas JA, RoyoDachary P, Ramírez Rodríguez JM, AguilellaDiago V, Martínez Díez M. Prevalence of anal diseases after Scopinaro’s biliopancreatic bypass for super-obese patients. Cir Esp. 2008;84(3):132–7. 18783671 10.1016/s0009-739x(08)72154-7

[16] Vanella S, Brisinda G, Marniga G, Crocco A, Bianco G, Maria G. Botulinum toxin for chronic anal fissure after biliopancreatic diversion for morbid obesity. World J Gastroenterol. 2012;18(10):1021–7. 22416176 10.3748/wjg.v18.i10.1021 PMC3296975

[17] Salgado-Nesme N, Santes O, Trejo-Ávila M, Morales-Maza J, Patiño-Gómez T, Solórzano-Vicuña D, et al. Incidence of benign anal diseases after bariatric surgery. Obes Med. 2020;20:100306.

[18] De Luca M, Angrisani L, Himpens J, Busetto L, Scopinaro N, Weiner R, et al. Indications for surgery for obesity and weight-related diseases: position statements from the International Federation for the Surgery of Obesity and Metabolic Disorders (IFSO). Obes Surg. 2016;26(8):1659–96. 27412673 10.1007/s11695-016-2271-4 PMC6037181

[19] Salminen P, Grönroos S, Helmiö M, Hurme S, Juuti A, Juusela R, et al. Effect of laparoscopic sleeve gastrectomy vs Roux-en-Y gastric bypass on weight loss, comorbidities, and reflux at 10 years in adult patients with obesity: the SLEEVEPASS randomized clinical trial. JAMA Surg. 2022;157(8):656–66. 35731535 10.1001/jamasurg.2022.2229 PMC9218929

[20] García-Redondo MF-M M, Rubio-Gil F, Belda-Lozano R, Ruiz-Pardo J, Sánchez-Fuentes P, Vidaña-Márquez E, et al. Enfermedad anal en pacientes candidatos a cirugía bariátrica: estudio descriptivo. Bariátrica Metabólica Ibero-Americana. 2023;13(1.3):3837–43.

[21] Huang J, Gui Y, Qin H, Xie Y. Causal association between adiposity and hemorrhoids: a Mendelian randomization study. Front Med. 2023;10:1229925. 10.3389/fmed.2023.1229925 PMC10587414 37869154

[22] Riss S, Weiser FA, Riss T, Schwameis K, Mittlböck M, Stift A. Haemorrhoids and quality of life. Colorectal Dis. 2011;13(4):e48-52. 20977590 10.1111/j.1463-1318.2010.02480.x

[23] Owen HA, Buchanan GN, Schizas A, Cohen R, Williams AB. Quality of life with anal fistula. Ann R Coll Surg Engl. 2016;98(5):334–8. 27087327 10.1308/rcsann.2016.0136 PMC5227050

