# Supplementary material for: High Incidence of Benign Perianal Disorders After Sleeve Gastrectomy and One Anastomosis Gastric Bypass
Source: Obes Surg. 2025 Oct 18;35(12):5256–63. doi: 10.1007/s11695-025-08342-1 (PMC12722501; doi:10.1007/s11695-025-08342-1)
Supplement: Supplementary file 1 — Supplementary file1 (DOCX 15 KB) [file 11695_2025_8342_MOESM1_ESM.docx]

**Appendix**

**1. What Is your year of birth?**

**2. Sex:** Male/Female.

**2. Any prior medical history of:**
**A. Obstructive Sleep Apnea**: Yes/No.
**B. Diabetes Mellitus:** Yes/No.
**C. Hypertension:** Yes/No.

**3. Preoperative weight:**

**4. Preoperative heigh:**

**5. Post operative weight:**

**6. Surgery type:**

**7. Revisional surgery**: Yes/No.

**8. Date of surgery:**

**9. Did you experience any change in bowel movements pattern after the surgery?**
A. More frequent bowel movements.
B. Less frequent bowel movements.
C. Hard/Dry stools.
D. Loose/Soft stools.
E. No changes in bowel habits.

**10. Number of bowel movements per day:**

**11A. Did you have any previous peri anal disease before the surgery?**
A. No.
B. Yes, not specified.
C. Anal fissure.
D. Hemorrhoids.
E. Peri anal abscess or fistula.
F. Rectal prolapse.
G. Fecal incontinence.

**11B. If the patient answered yes on section 11A: Did the prior peri anal disease worsen post-operatively?**
A. Yes.
B. No.

**12. For patients without a history of prior peri anal disease, did you develop a new peri anal disorder after the surgery?**
A. No.
B. Yes, not specified.
C. Anal fissure.
D. Hemorrhoids.
E. Peri anal abscess or fistula.
F. Rectal prolapse.
G. Fecal incontinence.

**13. Did you consult a healthcare professional for peri anal symptoms?**A. Yes.
B. No.

**14. Did you receive medical treatment for the new/worsening peri anal disorder?**A. No.
B. Stool softeners.
C. Topical anal fissure cream.
D. Topical hemorrhoids cream.
E. Antibiotics.

**15. Did you undergo any surgical intervention for new/worsening peri anal disorder?**
A. No.
B. Sphincterotomy.
C. Hemorrhoidectomy.
D. Hemorrhoids banding.
E. Seton drainage.
F. Abscess drainage.
G. Fistulotomy.
H. Botulinum A toxin injection for anal fissures.
I. Other.

**16. Are you currently suffering from the new/worsening peri anal disorder?**
A. Yes.
B. No.

**17. Were you aware that bariatric surgeries might lead to the development of peri anal disorders or exacerbate existing peri anal disorders?**
A. Yes.
B. No.

**18. Would you advise bariatric surgeries for other people suffering from obesity?**
A. Yes.
B. No.
